# Acknowledgment to Reviewers of *Foods* in 2021

**DOI:** 10.3390/foods11030367

**Published:** 2022-01-27

**Authors:** Foods Editorial Office

**Affiliations:** MDPI AG, St. Alban-Anlage 66, 4052 Basel, Switzerland

Rigorous peer-reviews are the basis of high-quality academic publishing. Thanks to the great efforts of our reviewers, *Foods* was able to maintain its standards for the high quality of its published papers. Thanks to the contribution of our reviewers, in 2021, the median time to first decision was 17 days and the median time to publication was 37 days. The editors would like to extend their gratitude and recognition to the following reviewers for their precious time and dedication, regardless of whether the papers they reviewed were finally published:
A. AruannoJoanna Zochowska-KujawskaAa Haeruman AzamJoao Carlos Gomes NetoAazza SmailJoão CotasAbdelouahed KhalilJoão DiasAbdelsamed ElshamyJoao F. ManoAbderrahmane Ait KaddourJoao Filipe Alberto LopesAbdolreza KharaghaniJoão Guilherme De Moraes PontesAbdul RohmanJoão Henrique Ghilardi LagoAbdul-Sattar NizamiJoão M. AlmeidaAbhiram ArunkumarJoao Ramalho-SantosAbiodun OmotayoJoaquim Calvo LermaAbolfazl MohebbiJoaquín Bautista-GallegoAbu Khayer Md. Muktadirul Bari ChowdhuryJoaquin NavarroAchille SchiavoneJoaquina PinheiroAchyut AdhikariJoel WeadgeAdam EkielskiJoerg KoenigstorferAdam FigielJogeir ToppeAdam KowalczykJohannes NovakAdam Le-GresleyJohannes StraussAdam LesnerJohn ArnasonAdam PacławskiJohn CarragherAdam T. SuttonJohn M. ThompsonAdamantini ParaskevopoulouJohn SamelisAdela AbellánJohn SnyderAdela Mora-GutierrezJohn-Lewis Zinia ZaukuuAdele PapettiJolanta GawalekAdil Malik NawazJolanta KowalskaAdil MouahidJolanta MierzejewskaAdina SantanaJolanta Orzelska-GórkaAdis PuškaJolanta RolaAdnan A. IsmaielJonathan KershawAdnane RemmalJonathan RoquesAdolfo PerrottaJonathon OlesbergAdrian AntosikJoong-Hyuck AuhAdrian AugustyniakJoon-Kwan MoonAdrian Krzysztof AntosikJoon-Young JunAdrián MatencioJordi RiuAdrián RabadánJörg EhlbeckAdriana ArigòJorge Alberto Sanchéz-BurgosAdriana Burlea-SchiopoiuJorge Álvarez-CervantesADRIANA DABIJAJorge Caceres-GianniAdriana FarahJorge Del RealAdriana HernándezJorge FreitasAdriana ŁobaczJorge LagoAdriana MikaJorge Moreno-FernandezAdriana SkendiJorge PereiraAdriana TrifanJorge PovedaAdriano Costa De CamargoJorge ReinheimerAdriano Gomes Da CruzJørgen LerfallAera JangJosé A. González-PérezÁfrica SanchizJosé A. Lopes-Da-SilvaAfzal HussainJosé A. LupiáñezAgapi DimaJose Angel Pérez-AlvarezAgata AntoniewskaJosé António CoutoAgata BiadałaJosé Antonio Curiel GámizAgata CzyżowskaJosé António De Oliveira E SilvaAgata FabiszewskaJosé Antonio Fernández-LópezAgata GórskaJose Antonio MolerAgata LeszczukJose BernalAgata MarzecJosé Blanco SalasAgata Wojciechowicz-BudziszJosé Carlos AndradeAgata ZnamirowskaJose Carlos Jimenez-LopezÁgnes BónaJose E Aguilar-ToaláAgnes WeissJose Emanuel Loeza AlcocerAgnieszka BartoszekJosé Francisco Segura PlazaAgnieszka BazylkoJosé L. RamblaAgnieszka BiałekJose Luis PérezAgnieszka CiurzyńskaJosé Luis Rodríguez GutiérrezAgnieszka Ewa Wia̧cekJosé Luis TodolíAgnieszka GalantyJose M. LorenzoAgnieszka GrzegorczykJosé M. OliveiraAgnieszka KicelJosé Manuel AguilarAgnieszka KitaJosé Manuel BenitoAgnieszka KorgaJosé Manuel CruzAgnieszka LudwiczakJosé Manuel Moreno-RojasAgnieszka MakowskaJosé María GarcíaAgnieszka Nawirska-OlszańskaJose María Rodríguez-CallejaAgnieszka NawrockaJose Martin Ramos-DiazAgnieszka NowakJose MendiolaAgnieszka Olejnik-SchmidtJosé Miguel Bastías MontesAgnieszka OrkuszJosé Miguel GuzmánAgnieszka RichertJosé Pérez NavarroAgnieszka SmalecJosé PinelaAgnieszka SzewczykJosé PiornosAgnieszka ViapianaJose Ros-LisAgustín ArandaJosé Segura-PlazaAgustín AriñoJose Sousa CamaraAgustín Llopis-GonzálezJosé Sousa CâmaraAGUSTÍN V. RUIZ VEGAJosef KameníkAhmad Al-MrabehJosep JulveAhmed A. SalehJoseph DumplerAhmed A. ZakyJoseph G. SebranekAhmed M. MustafaJoseph KannerAhmed M. SayedJoseph MarinoAhmed NafisJoseph SmilanickAhmed R. A. HammamJosias MeribAibo WuJosip ŠimunovićAikaterini ArgyropoulouJosivanda P. GomesAili JiangJosué DelgadoAiliang ChenJovana KojićAina García-GarcíaJovana KovacevicAiry Gras MasJovanov PavleAjay KumarJózsef FelföldiAjay Kumar YagatiJózsef PoppAjit K. MahapatraJuan A. CentenoAkihiro HandaJuan B. CalancheAkira IshidaJuan Carlos Cuevas BernardinoAkira WanikawaJuan Carlos López-LinaresÁkos BodnárJuan CórdobaAlain D'AstousJuan De Dios Alché RamírezAlain DeloireJuan García-DíezAlain Le-BailJuan García-ReyesAlam ZebJuan José Martínez NicolasAlbert BordonsJuan Luis Pérez Pérez BernalAlbert Linton CharlesJuan Luis ValenzuelaAlbert Ribas-AgustíJuan Monzó-CabreraAlberto AngioniJuan MunizAlberto BrugiapagliaJuan Pablo Fernández-TrujilloAlberto FalcóJuan PolariAlberto FioreJuan Rubio AlonsoAlberto GallegosJuanas FriasAlberto GarréJuan-García AnaAlberto Horcada IbáñezJudit KrischAlberto Michele FelicettiJudson GrubbsAlberto NiccolaiJudy CunninghamAlberto NuñezJuei-Tang ChengAlberto RitieniJuha TaavelaAlberto RomeroJuhani PirhonenAlberto ValdésJui-Yang LaiAldo TavaJulia LowAldona SobotaJulia SchwarzkopfAlejandra Bermúdez-OriaJulia Wojciechowska-SolisAlejandra Garcia-AlonsoJulie BowerAlejandro Díaz-MorcilloJulie ReganAlejandro Fernández ArteagaJulien ParinetAlejandro Garrido-MaestuJuliet R. RobertsAlejandro Javier ParedesJulio Alvarez PittiAlejandro José Laguna SanzJulio Nogales-BuenoAlejandro Rivas SolerJulita RegulaAlejandro Téllez-JuradoJung KwonAleksandar Z. FištešJung Sun HongAleksandar Ž. KostićJungmoo HuhAleksander PoredaJunheon KimAleksandra Duda-ChodakJunho ChungAleksandra KocotJun-ichi KadokawaAleksandra KołotaJunjie YiAleksandra KristoJunli XuAleksandra KrolickaJun-Sang HamAleksandra MišanJunseong ParkAleksandra PawlaczykJunzeng ZhangAleksandra SzcześJurica ŽučkoAleksandra SzydlowskaJuristo FonolláAleksandra Szydłowska-CzerniakJuscelino TovarAleksandra WilczyńskaJustin RenaudAleksandra ZambrowiczJustyna BochnakAlen DzidicJustyna Dobrowolska-IwanekAlena SalákováJustyna GodosAleš KuharJustyna GrabskaAles RajchlJustyna LiberaAlessandra BendiniJustyna Płotka-WasylkaAlessandra BiancolilloJustyna Rosicka-KaczmarekAlessandra DalmassoJustyna ŻulewskaAlessandra De BrunoJyh-fei LiaoAlessandra GuidiJyrki VirtanenAlessandra JagerKacie HoAlessandra MartiKai Min YangAlessandra SpennatoKai Victor HansenAlessandro BonadonnaKai ZhangAlessandro FeisKairi KivirandAlessandro FeolaKajetan MüllerAlessandro GrinzatoKalidas ShettyAlessandro MaugeriKalina Maja Sikorska-ZimnyAlessandro ZambonKaliramesh SiliveruAlessia TrimignoKaliyan BarathikannanAlessia TropeaKalu Kapuge Asanka SanjeewaAlessio AllegraKalyan BarmanAlessio CappelliKamel MsaadaAlessio CavicchiKamil Krzysztof RomanAlessio ScarafoniKamil PiwowarekAlex M. PierceKamil WitaszekAlex ManicardiKamila RachwalAlexander BetekhtinKamila Rybczyńska-TkaczykAlexander G. DvoretskyKamila Stokowa-SołtysAlexander K. GoroncyKamila ZdenkovaAlexander M. ZakharenkoKandasamy SaravanakumarAlexander Nesterov-MuellerKandhasamy SowndhararajanAlexander OsmolovskiyKandi SridharAlexander SchaussKaneda HirotakaAlexander SchjøllKang Lan TeeAlexander TsouknidasKang-Mo KuAlexander TyakhtKannappan ArunachalamAlexandra FurchKaoru KohyamaAlexandra J. MayhewKarel KroftaAlexandra LianouKari KopraAlexandra MezitiKarim AllafAlexandra MouraKarin SchwarzAlexandra MüllerKarin WendinAlexandre LamasKarl R. MatthewsAlexandros MavrommatisKarna RamachandraiahAlexia N. GloessKarol SteckiewiczAlexia PradesKarolina A. Wojtunik-KuleszaAlfio SpinaKarolina Brkić BubolaAlfonso ClementeKarolina JakubczykAlfonso Robles MedinaKarolina Matej-ŁukowiczAlfredo MiccheliKarolina PyciaAlfredo MontañoKarolina SzulcAlfredo PalopKarolina TkaczAli KhoddamiKarolina WieszczyckaAli Sedaghat DoostKarolina WójciakAlice LeeKaroly HebergerAlice VilelaKashif AmeerAlicia AguilarKassiani MellouAlicja PonderKata GalicAlida SorrentinoKatalin LányiAlina AvanesyanKatarina MosnackovaAlina MagdasKatarzyna Bober - MajnuszAlina PykaKatarzyna CzaczykAlina Violeta UrsuKatarzyna DettlaffAlisha PrasadKatarzyna Dybka-StępieńAlison HarmonKatarzyna JandaAlison R. GerkenKatarzyna Kajak-SiemaszkoAlissa NoldenKatarzyna KowalskaAlla SplichalovaKatarzyna Małolepsza-JarmołowskaAllan PhilippeKatarzyna Marciniak-LukasiakAlma D. Alarcon-RojoKatarzyna Marciniak-ŁukasiakAlma Elizabeth Cruz GuerreroKatarzyna Neffe-SkocińskaAlvaro Fernandez OchoaKatarzyna PobiegaAlyssa Hidalgo VidalKatarzyna RafińskaAmali U. AlahakoonKatarzyna RatuszAmalia ConteKatarzyna RubinowskaAmalia PiscopoKatarzyna RybakAmanda Dupas De MatosKatarzyna SkrzypczakAmanda KinchlaKatarzyna ŚliżewskaAmanda M. WindsorKatarzyna SosnowskaAmanda SiegelKatarzyna Sułkowska-ZiajaAmanda StewartKatarzyna ŚwiąderAmaury Taboada-RodríguezKatarzyna Sykłowska-BaranekAmedeo LonardoKatarzyna WaszkowiakAmel TaibiKatarzyna WińskaAmelia DelgadoKatarzyna WłodarskaAmélie BancKatarzyna ZawadaAmin Mousavi KhaneghahKaterina TzimaAmir GhandiKatherine WitrickAmit JaiswalKathia Jimenez MonroyAmit SinghKathleen KevanyAmmu K. RadhakrishnanKathleen OehlkeAmna SaharKathrine BakAmparo GonçalvesKatia Laura SidaliAna Belén FlórezKatia PetroniAna Belén RoperoKatja KröllerAna Beltrán SanahujaKatja ZmitekAna Bucić-KojićKatya CarboneAna Cristina Agulheiro SantosKavindra Kumar KesariAna Cristina CorreiaKavita SharmaAna Cristina FigueiredoKavitha PathakotiAna Cristina Sanchez-GimenoKaWang LiAna FonsecaKazuhiro HoriAna G. PérezKazuko Ishikawa-TakataAna Gomes-BispoKazuo MiyashitaAna Henriques MotaKazuya MorimatsuAna HerediaKebede Manjur GebruAna HranilovicKebede Taye DestaAna I. CarrapisoKeehoon LeeAna Isabel Álvarez-MercadoKeiji JindoAna Isabel CostaKeisuke SasakiAna Isabel SanchoKeith WarrinerAna JeromelKelly GettyAna Jiménez-AraujoKelly GeyskensAna Jurinjak TusekKelly Johana Figueroa LópezAna KaicKelsey GabelAna L. AmaroKelvin Kim Tha GohAna Lúcia Da Silva OliveiraKen-ichiro MinatoAna M. M. GonçalvesKenichiro NakajimaAna M Vivar-QuintanaKen-ichiro ShimadaAna Maria Gomez NeoKenji MaehashiAna María Jiménez CarveloKenneth J. PrusaAna Maria SoaresKenneth W. McMillinAna Marija Jagatić KorenikaKenshi WatanabeAna Mendes-FerreiraKerensa BroersenAna Paula DuarteKerri GehringAna RoldánKerry WilkinsonAna Sanches SilvaKeshun LiuAna Sanchez SilvaKevin HogeveenAna Sofia Pereira MoreiraKevin KantonoAna V. González-De-PeredoKevin Mis SolvalAnabela RaymundoKeyu TaoAnaberta Cardador-MartínezKhanh-Hoang NguyenAnamaria PopKhaoula KhwaldiaAnamaria SdrobisKi Hwan ParkAnanta Prasad ArukhaKi-Baek JeongAnastasia BadekaKiharu IgarashiAnastasia HyrinaKim Hyeong SangAnastasia KtenioudakiKim JanssensAnastasia P. GalanopoulouKim-Yen Phan-ThienAnastasios AlatzasKinga DrzewieckaAnastasios NikolaouKiri McCombAnatoly P. SobolevKirill TkachenkoAnca DoceaKirsten BrandtAndras BittsanszkyKishu RanjanAndrea AnnibalKit Leong CheongAndrea AntonelliKiyonori KawasakiAndrea ArmaniKlara KraljićAndrea ArsiccioKlaus G. GrunertAndrea BoscoKlaus-Peter GötzAndrea BragaglioKoh HashidaAndrea BrandoliniKoji HosomiAndrea CabidduKolbrun SveinsdottirAndrea CarpentieriKolbrún SveinsdóttirAndrea CerratoKonstadinos MattasAndrea FulgioneKonstantin V. MoiseenkoAndrea GarmynKonstantina ArgyriAndrea Giuseppe MaugeriKonstantinos ArsenopoulosAndrea MarchettiKonstantinos GerasopoulosAndrea MarchiniKonstantinos PetrotosAndrea McWhorterKonstantinos PolymerosAndrea NatolinoKosmas NathanailidesAndrea PautzKosuke MotokiAndrea PorzelKoushik AdhikariAndrea R. TeufelbergerKovačević Danijela BursaćAndrea RagusaKowalska MałgorzataAndrea SalvoKresimir MastanjevicAndrea SummerKrešimir MastanjevićAndreana PexaraKriskamol Na JomAndreas EisenreichKristina KljakAndreas ExnerKristina KukurovaAndreas WinklerKristina MastanjevićAndreea RatiuKristina StarčevićAndreia FreitasKristl JanjaAndrej OvcaKrystyna GutkowskaAndres Barajas-SolanoKrystyna RejmanAndres David RomanKrystyna Szymandera-BuszkaAndrew Bamidele FalowoKrzysztof B. BećAndrew ByrneKrzysztof DasiewiczAndrew DesboisKrzysztof GornickiAndrew G. GehringKrzysztof GorynskiAndrew OzgaKrzysztof KilianAndrew RosenthalKrzysztof PrzybyłAndrey A. TyuftinKrzysztof TutajAndri FrediansyahKsenija MarkovićAndrius GarbarasKuan-Chen ChengAndriy SynytsyaKumar LamaAndrzej KurendaKuo-Chiang HsuAndrzej WasikKwan Seob ShimAnees Ahmed KhalilKwang-Ming LiuAnet Rezek JambrakKwang-Ok KimAneta BrodziakKyrre RickertsenAneta JastrzębskaL. OteroAneta KrogulskaLachlan SchwarzAneta PaterLaetitia L.S. Canabady-RochelleAnette E. BuykenLaetitia ThéronÁngel Calín-SánchezLaila M. MoujirAngel Carbonell-BarachinaLajnaf RouaAngel CobosLalit GolaniAngela BisioLamin KassamaÁngela Bravo-NúñezLanfranco ConteAngela CostaLara CostantiniAngela DambrosioLara ManyesAngela Di PintoLara Saftić MartinovićAngela FaddaLarbi RhaziÁngela G. SolaesaLászló BaranyaiAngela Tellez-LanceLászló ÓzsváriAngela ZappiaLászló SiposAngela ZinnaiLaura CampanoneAngélica Espinoza-OrtegaLaura CasulaAngelica Quintero-FlorezLaura CerqueiraAngélica Villarruel-LópezLaura GazzaAngeliki KatsafadouLaura Higueras ContrerasAngelo Antonio D’ArchivioLaura JefferiesAngelo CichelliLaura LagunaAngelo FacchianoLaura LalondeÂngelo LuísLaura Martínez-CarrascoAngelo Maria GiuffrèLaura MoralesAngelos SikalidisLaura MorelloAni RadonićLaura PascualAnika SinghLaura RighettiAnil K. VermaLaura RomanAnil VermaLaura RossiAnita JakobsenLaura SelanAnita PichlerLaura StanAnita RáczLaura ToribioAnja Steffen-HeinsLaura Torres-ColladoAnka Trajkovska PetkoskaLaura Vázquez-AraújoAnket SharmaLaurel L DunnAnna AdámkováLaurent DufosséAnna Berthold-PlutaLauro MeloAnna Bilska-WilkoszLavanya GoodlaAnna DąbrowskaLavanya ReddivariAnna Di SalleLavinia Florina CălinoiuAnna DiowkszLeacady SalibaAnna DunayLeanne YoungAnna FlorowskaLeao Martins José ManuelAnna GajdaLech AdamczakAnna Gramza – MichałowskaLecturer Carmen Rodica PopAnna GreppiLei Hong TaoAnna IwaniakLei WangAnna Jarosz-WilkołazkaLélia ChambelAnna JelińskaLelia VoineaAnna KaczmarekLene Duedahl-OlesenAnna Kamińska-DwórznickaLenka KouřimskáAnna Kostecka-GugalaLenka KubicovaAnna KotLenka LanghansovaAnna KulmaLeo NankervisAnna LanteLeonard KoolmanAnna LenziLeonardo CaputoAnna M. EhlersLeonardo CasiniAnna M. F. MarinoLeonardo Rios SolisAnna MagnanoLeonardo SepúlvedaAnna Maria KotLeonel PereiraAnna Maria PosadinoLesław JuszczakAnna MarouškováLeslie D. BourquinAnna MichalskaLeszek MoscickiAnna MitrakiLeszek SzablewskiAnna MottolaLeticia MoraAnna NowakLeticia Xochitl Lopez-MartinezAnna OgrodowczykLetizia BrescianiAnna OkońLia Tânia J DinisAnna OniszczukLiane WagnerAnna PintoLiang RongrongAnna PodsedekLiang-Yu ChenAnna PtaszekLibo TanAnna PuigLibor CervenkaAnna RealeLibor KalhotkaAnna Rita BiliaLidia GaffkeAnna Rzepecka-StojkoLídia PinheiroAnna SadowskaLidia Stasiak-RóżańskaAnna Sadowska-RociekLidija JakobekAnna SandionigiLidija KozačinskiAnna ShchennikovaLidija SvecnjakAnna SikoraLihan HuangAnna StepienLih-Shiuh LaiAnna Szczerba-TurekLihua HouAnna VikulinaLijing FangAnna Winiarska-MieczanLili HeAnna Wondołowska-GrabowskaLili JiaAnna ŻbikowskaLili MatsAnna Zimoch-KorzyckaLilian Regina Barros MariuttiAnna ŻołnierczykLiliana G. FidalgoAnnabella TramiceLiming YeAnnalisa ChiavaroliLin ChunyaAnnalisa D’ArcoLina CossignaniAnnalisa De BoniLinards KlavinsAnnalisa MaiettiLinda ScobieAnnalisa MentanaLingyan KongAnnalisa RomanoLinus Mabughi NyiwulAnnalisa TassoniLionel BoillereauxAnnamaria RanieriLisa L. DeanAnnarita PanighelLisard Iglesias-CarresAnnchen MielmannLisbeth MehliAnne Dahl LassenLise Nistrup JørgensenAnne DuconseilleLisetta GhiselliAnne Maria MullenLiubov SkrypnikAnne RiederLiudmila NadtochiiAnne-Claire Mitaine-OfferLoïc LeclercqAnne-Gabrielle MathotLongyan ZhaoAnne-Grethe JohansenLoong-Tak LimAnne-Marie HermanssonLoredana DumitrascuAnne-Marie Pensé-LhéritierLorena MartínezAnnette KuehnLorenzo CecchiAnnette RompelLorenzo GarciaAnnie HillLorenzo GuzzettiAnoma ChandrasekaraLorenzo MorelliAnshu AlokLorenzo PalombiAnshu RastogiLorenzo ZanellaAnte LončarićLoris PintoAnte PrkićLouis Kuoping ChaoAnthia MatsakidouLoulouda BosneaAnthimia BatrinouLourdes Casas CardosoAnthony FardetLovedeep KaurAntía González PereiraLowell D. KispertAntimo Di MaroLuana Beatriz Dos Santos NascimentoAntje RisiusLuc IngenbleekAntonella ArestaLuc SaulnierAntonella Caterina BocciaLuca AmbrosinoAntonella CostaLuca BenvenutiAntonella CostantiniLuca CamponeAntonella Della MalvaLuca DellafioraAntonella FaisLuca FortiAntonella PasqualoneLuca IseppiAntonella ProfumoLuca LanotteAntonella VerzeraLuca M. ChiesaAntoni SzumnyLuca NunziataAntonia BrunoLuca PellegrinoAntonia LaiLuca PennisiAntonia M. Jiménez-MonrealLuca RosciniAntonia TerpouLuca SecondiAntonietta BaianoLuca SettanniAntonin KanaLuca TricaricoAntonina GiammancoLucas Dos Santos DiasAntonino NizzaLucia De CastelliAntonino SpanuLucia MaddaloniAntónio AbreuLucía Olmo-GarcíaAntonio Amores ArrochaLucia PanzellaAntonio AsciutoLucia ParafatiAntonio Benítez-CabelloLucia SepeAntonio BevilacquaLucia VanniniAntonio BlancoLucian CopoloviciAntonio CelliniLuciana Calheiros GomesAntonio CillaLuciana De VeroAntonio ColantuonoLuciana TorquatiAntonio D. Rodriguez-LopezLuciano CinquantaAntonio Di MartinoLuciano PiergiovanniAntonio Diogo Silva VieiraLuciano PinottiAntonio Garrido-FernándezLucie HasoňováAntónio Garrido-FernándezLucimara G. De La TorreAntonio GiovinoLucio CapursoAntonio Gonzalez-CasadoLucrecia Carrera QuintanarAntonio Herrera-HerreraLucrezia SergioAntonio J. Pérez-LópezLucrezia VolpiAntonio J. Sanchez-SalmeronLuigi BrunettiAntonio José Lozano GuerreroLuigi De MasiAntonio Lama-MuñozLuigi IannettiAntonio López GómezLuigi MenghiniAntónio M. PeresLuigia PazzagliAntonio MarínLuigino BarisanAntonio Martínez LópezLuís AbrunhosaAntonio Martinez-AbadLuis Alfonso Trujillo-CayadoAntonio MincioneLuís C. N. Da SilvaAntónio NogueiraLuis Federico CasassaAntónio P. L. MartinsLuís Filipe RibeiroAntonio PanebiancoLuis J. BastarracheaAntonio PezoneLuis M. Carrillo-LópezAntonio ValentoniLuis NogueraAntonio ZuorroLuís PatarataAntonios E. KoutelidakisLuís PintoAntti KnaapilaLuis Puente-DíazAnubhav Pratap SinghLuis RodrigoApinun KanpiengjaiLuis TejadaAránzazu MedieroLuisa AlbaranoArash MoeiniLuísa BarreiraArchimede RotondoLuisa CustodioArianna BinelloLuisa PellegrinoArianna DondiLuisa PozzoArianna LovatoLuísa SerafimArianna RicciLuisa TorriAriel FontanaLuis-Alberto Casado-ArandaArijit NathLuiz Carlos FilhoArio De MarcoLuiz SilvaAris GiannakasLukáš KučeraAristeidis TsagkarisLukas SnopekArkadii ArinsteinŁukasz GierzArkadiusz ArtyszakŁukasz KrystArkadiusz MatwijczukŁukasz P. HalińskiArmando Alexei RodríguezŁukasz SatołaArmando Perez-CuetoŁukasz SeczykArmando ZarrelliŁukasz SęczykArmandodoriano BiancoŁukasz SzeleszczukArmida Sánchez-EscalanteLuminita CrisanArne DuinkerLuna Maslov BandićArnoldo LopezLu-Sheng HsiehArokia Mariyadoss VijayaanandLutz GrossmannArokia Mariyadoss VijayanandLuud J.W.J. GilissenÁron TörökLyudmila I RachekArrigo CiceroM. Angeles RomeroArtur GryszkinM. Azizur RahmanArtur J. MartinsM. Elvira López-CaballeroArtur Jorge Araújo Magalhães RibeiroM. Eugenia TornadijoArtur JóźwikM. Graça M. Graça DiasArtur MartinsM. Leonor NunesArtur RzeżutkaM. Luisa Escudero-GileteArtur SzwengielM. Sonia FreireArtur WiktorM. Teresa SanchoArturo Hardisson De La TorreM.A. Del NobileArulkumar NagappanMaaria KortesniemiAshenafi F. BeyiMabel BladesAshish RawsonMacarena FarcuhAshutosh BahugunaMaciej BalawejderAsker BrejnrodMaciej GuzikAsraful AlamMaciej JarzebskiAswin VerhoevenMaciej KluzAthanasia PrintzaMaciej NastajAthanasios K AnagnostopoulosMaciej OziembłowskiAthanasios MallouchosMaciej StrzemskiAthanassios AlexopoulosMaciej WójcikAthina NtzimaniMack ShelleyAtte Von WrightMackenzie HannumAttila GereMadeleine GranvikAttila JámborMadoc SheehanAttilio MordentiMafalda AlmeidaAudrey TierneyMagali ChohanAurora SilvaMagdalena DadanAurora VassanelliMagdalena Frej-MądrzakAusilia GrassiMagdalena Jeszka-SkowronAušra RūtelionėMagdalena Klimek-OchabAutumn MarshallMagdalena KrystyjanAvinash KadamMagdalena LigorAvtar MatharuMagdalena Makarewicz-WujecAwang MaharijayaMagdalena Martínez-CañameroAyanka WijayawardenaMagdalena Orczyk-PawiłowiczAylin SahinMagdalena PizońAzarmidokht Gholamipour-ShiraziMagdalena Polak-BereckaAzzurra AnnunziataMagdalena RudzińskaB. B. DzantievMagdalena Słowik BorowiecB. WróblewskaMagdalena SobiesiakBabar MurtazaMagdalena Wirkowska-WojdyłaBachir BenarbaMagdalena WronaBalamuralikrishnan BalasubramanianMagdalena ZalewskaBalasubramani Subramani ParanthamanMagdalena ZdanowiczBalkrishna GhimireMags CrumlishBaltasar MayoMahboob AlamBamidele RaheemMahmoud El-MaghrabeyBaolei JiaMahmoud F. SeleimanBarbara BigliardiMahsa MajzoobiBarbara BrunettiMaja BenkovićBarbara CampisiMaja DražićBarbara CuomoMaja Jazvinšćak JembrekBarbara GiussaniMaja Jukic SpikaBarbara LanzaMaja KozarskiBarbara Nieradko-IwanickaMaja Krstić RistivojevićBárbara Nieva-EchevarríaMaja MolnarBarbara SawickaMajid ShakeriBarbara SchalchMalcolm RileyBarbara SocasMalgorzata DobrzynskaBarbara SpolaoreMalgorzata DzuganBarbara TiozzoMałgorzata DżuganBarry ParsonsMałgorzata Ewa DrywieńBartłomiej KostMalgorzata GajewskaBartosz KruszewskiMałgorzata GniewoszBartosz SołowiejMałgorzata GrembeckaBartosz SzulczyńskiMałgorzata GumiennaBasharat Nabi DarMałgorzata Kalemba-DrożdżBeata DruzynskaMałgorzata Kapelko-ŻeberskaBeata DrużyńskaMałgorzata KarwowskaBeata KuczyńskaMałgorzata KołodziejczakBeata MikołajczakMalgorzata KorzeniowskaBeatrice FalcinelliMalgorzata KosteckaBeatriz GullonMałgorzata KozyraBeatriz Sevilla-MoránMałgorzata ŁabańskaBeatriz Vazquez BeldaMałgorzata Lasik-KurdyśBegoña PaneaMałgorzata MajcherBelem BarbosaMalgorzata NowackaBelinda López-GalánMałgorzata NowackaBenedetta BottariMałgorzata PawlosBenedetta MargiottaMałgorzata PiecykBenedikt M. MortzfeldMałgorzata PiotrowskaBeng Fye LauMałgorzata RutkowskaBeniamino Cenci GogaMałgorzata SobczakBeniamino T. Cenci-GogaMałgorzata StarowiczBenjamin CarbonnierMałgorzata ŚwiątkiewiczBenjamin G. PolkinghorneMałgorzata SzczepanekBenjamin M. BohrerMałgorzata Szultka-MłyńskaBenjamin OrsburnMałgorzata TabaszewskaBenno WeigmannMałgorzata WroniakBenoit ChenaisMałgorzata WronkowskaBenoit CudennecMalgorzata Zakłos-SzydaBenoît IgneMałgorzata ZiarnoBernadette TelekyMallique QaderBernard ChenMalshani L. PathirathnaBernard FayeMalvina OrkoulaBernd BonnländerMamen Cuéllar-PadillaBernd HitzmannMamoru IsemuraBernd W. SpurManel CampsBert DriessenManfred EggersdorferBert WeijtersManfred GrossmannBerta Baca-BocanegraMan-Gi ChoBertrand MatthäusMani NaikerBeth MallardManlio BaccoBettina WolfManohar Prasad BhandariBhagyashree N. SwargeMansour SobehBiagio FallicoManuel Malfeito-FerreiraBianca Chieregato ManigliaManuel Moya-VilarBianca R. AlbuquerqueManuel Recio-MenéndezBiancamaria SenizzaManuel SánchezBianka GrunowManuela AmorimBiao ZengManuela CorreiaBidisha SenguptaManuela GrimaldiBin WangManuela MariottiBingyin PengManuela OliveiraBinxiao FuManuela PeukertBirgit GeuekeManuela RolliniBirgit RumpoldMara BanovićBirthe P. Møller JespersenMarc PignitterBiserka RelicMarc RegierBlanaid WhiteMarcel KrzanBlanca Hernández-LedesmaMarcela SegundoBlaženka KosMarcelo BeckerBo PangMarcia EnglishBo WangMárcia VidigalBobbi PrittMarcin FrankowskiBogdan Păcularu-BuradaMarcin KurekBogdan SmykMarcin LukasiewiczBohdan LuhovyyMarcin ŁukasiewiczBojan ŠarkanjMarcin MitrusBojan ŽunarMarcin SchmidtBojana VoučkoMárcio RodriguesBo-Kang LiouMarco A. MurgiaBomi RyuMarco A. PalmaBonastre OlieteMarco BeccariaBożena KiczorowskaMarco BloklandBrad RidouttMarco BraviBrahim BchirMarco ConsumiBranca SilvaMarco DacremaBrandon RestrepoMarco EstiBranimir PavlićMarco FranzoiBranislav ŠojićMarco GobbiBranko PopovićMarco MangiagalliBranko SuvajdžićMarco MontemurroBrennen MillsMarco SantinBrian FarnetiMarcos FloresBrianne A. AltmannMarcus BaumannBrienna L. Anderson-CoughlinMarcus RohnkeBrigitta TóthMarek AljewiczBruce BernackiMarek BrabecBruce R. CooperMarek GancarzBruna CarbasMarek GaworskiBruno Melgar CastañedaMarek Kardas Marek KardasBuldo PatriziaMarek KieliszekByron WilliamsMarek KotowiczByung-Chun InMarek PieszkaC. H. ChouMarek VeckaC. Korcan AyataMargaret HarrisC. Leigh BroadhurstMargaret L. BritzC. M. BotelhoMargareta NymanCaleb AcquahMargherita AmentaCalogero DiBellaMargherita MarzoniCamila D. Madeira CamposMargherita PettinatoCamilla CattaneoMaría África Fernández-PriorCan EyupogluMaria Alessandra PaissoniCarine Delayre-OrthezMaría Ángela Burrell BustosCarla AlegriaMaria Angela PeritoCarla B. SchubigerMaría Ángeles Fernández-ZamudioCarla Busquets-CortésMaría Ángeles SantosCarla CavalloMaria Antonietta CiardielloCarla GentileMaría Asensio-RamosCarla NunesMaria AstolfiCarla PereiraMaria AtanassovaCarlo BoselliMaria Belén LinaresCarlo D'AscenziMaria C. FernandesCarlo GenoveseMaria C. GiannakourouCarlo Maria CarbonaroMaria CamposCarlos A. Ortega-ZúñigaMaria Carla CraveroCarlos Alberto De Almeida GadelhaMaria Carmo BarretoCarlos Bravo-DiazMaria Cecilia PuppoCarlos Dias PereiraMaria CefolaCarlos EscottMaria CermenoCarlos Gutierrez-MerinoMaria Ciudad-MuleroCarlos GuzmanMaria Concetta StranoCarlos Iván Delgado-NieblasMaria Consuelo Pina-PerezCarlos Martin-RiosMaria Cristina MessiaCarlos Palacios RiocerezoMaria D. MesaCarlos RibeiroMaria Da Graça Costa MiguelCarlos SabaterMaria DapkeviciusCarlos VasconcelosMaría De Cortes Sánchez-MataCarlotta GirominiMaría De Los Ángeles Ortega De La TorreCarme GüellMaria De Lurdes DapkeviciusCarmela GerardiMaria De MieriCarmela LamacchiaMaría Del Carmen PérezCarmela Maria MontoneMaría Del Mar CampoCarmelo CorsaroMaria DimopoulouCarmelo Maria MusarellaMaria Dolores GuillénCarmen BarbaMaria DzikućCarmen CuadradoMaría E. García-PastorCarmen GarcíaMaria E. M. AraujoCarmen Garcia-JaresMaria Eduarda PotesCarmen LiconMaria Eduardo FigueiraCarmen Licon-CanoMaria Elena LatinoCarmen MannucciMaria Eleni SakavitsiCarmen Rodríguez GarcíaMaria Elisabetta BaldassarreCarmen RubioMaría Elvira López-OlivaCarmen Vidal-CarouMaria Emilia JuanCarmine NegroMaría Esperanza ValdésCarmine RoccaMaria Fátima SilvaCarmine SummoMaria Federica TrombettaCarole CamarasaMaria Fernanda PessoaCarolina FloresMaría Figueiredo-GonzálezCarolina Muñoz-GonzálezMaria Fiorenza CaboniCaroline StrubMaria Fraga-CorralCarsten MüllerMaria Francesca CardoneCasilda NavarroMaria Francesca IuliettoCasimiro MantellMaria FranciosiniCatarina L. SilvaMaria Francisca Blasco LopezCaterina AgrimontiMaría Gabriela MerínCaterina MannaMaría GianelliCaterina MorciaMaria Grazia VolpeCaterina VillaMaria GulloCatherine ChanMaría Gutiérrez-SalcedoCatherine H. ScheinMaría Hortigón-VinagreCatherine KellyMaria Inês Sucupira MacielCatherine LacosteMaria J. AlcaldeCatherine M CookMaría J. CantalejoCatherine MacombeMaría Janeth Rodríguez-RoqueCatia MartinsMaria JeznachCatrin E TylMaria João BarrocaCatrin TylMaria João RamalhosaCecilia Mark-HerbertMaria João RodriguesCecilia NunesMaría José Beriáin ApesteguíaCeferino Carrera FernándezMaría José Muñoz-AlférezCelia C. G. SilvaMaria José SilvaCélia C. G. SilvaMaria Judite AlmeidaCem Okan ÖzerMaria KatsouliChaiyavat ChaiyasutMaria KyraleouChan Kyung KimMaria Lage-YustyChandrakala Aluganti NarasimhuluMaria LavillaChang ChenMaria Leonor NunesChang Joo LeeMaria Lucia Valeria De ChiaraChangqi LiuMaria Luisa BraungerChao-Hui FengMaria Luisa DettoriCharalampos KarantonisMaria Luísa SerralheiroCharalampos ProestosMaría M. Morales Suárez-VarelaCharis M. GalanakisMaria Madalena SobralCharles BrennanMaria MargalefCharles L. WilkinsMaria MartinCharles M. MacVeanMaria MartuscelliCharles Odilichukwu R. OkpalaMaria NobileCharles R. FrihartMaria PaciulliChen YangMaria Paola GermanòChen ZhouMaría Paz Aguilar-CaballosChiachung ChenMaría Pilar AlmajanoChia-Hung KuoMaria Pilar CortésChiara BeltramoMaria Pina ConcasChiara MonachesiMaria PiochiChiara MontanariMaria Raquel Marçal NataliChiara NadaiMaría Rocío Rodríguez ArcosChiara NedianiMaria RomoChieh-Yu LinMaria Ruiz MendezChih Yao HouMaría SerranoChih-Hsiang ChangMaría Soledad PratsChih-Hua TsengMaria Teresa Bertoldo PachecoChing-Hu ChungMaría Teresa Murillo-ArbizuChing-Kuo LeeMaría Teresa Sánchez BallestaChing-Yi ChenMaria Vazquez-HernandezChinmay SatamMaria Veronica Chandra-HioeChiranjeevi KorupalliMaria VitaleChitang HoMaria-Carmen Lopez De Las HazasChitrita DebRoyMariana María HuertaChorng-Liang PanMariana VeigaChris ArthurMarianna CaterinoChris AustinMarianna GiannoglouChris BearMarianna MartinelloChris BishopMarianna RaczykChris BurtMarianthi BasalekouChris DouvrisMaria-Paz RomeroChristian A. MuellerMaría-Pilar Sáenz-NavajasChristian AndrighettoMariarosaria MilosoChristian CoelhoMarie AlmingerChristian PhilippMarie BorkovcováChristian R. Encina-ZeladaMarie FilteauChristian RothMarie Laure FauconnierChristian SallesMarie WalshChristian SteuerMarie-Louise ScippoChristian VoglMarie-Pierre Ellies-OuryChristian Von WallbrunnMarietta FodorChristiane HilgerMarija Badanjak SabolovićChristianne Elisabete Da Costa RodriguesMarija CerjakChristina A. Mireles DewittMarija KindlChristina C. TamMarijana AčanskiChristina E. LarderMarijana Marek SarićChristine BoschMarijana PopovićChristine Feillet-CoudrayMarijana SakačChristine H. ScamanMarijana Zovko KončićChristine Robert-Da SilvaMarika Dello RussoChristophe BliardMarika LanzaChristopher BryantMarina Cano LaMadridChristopher GlassonMarina CreydtChristopher J. SmithMarina IsidoriChristopher TaylorMarina KostićChristos I. RumbosMarina P. ArrietaChristos K. MichailMarina P. Arrieta DillonChristos KontogiorgisMarina PadilhaChristos PappasMarina RussoChung BuiMarina SagnouChung-kuang LuMarina TomićChung-Tung J. LinMarina VillalonChunhong YuanMarine CouéChunki KimMarinela NutrizioChun-Kuang ShihMario AmatoChun-Yao YangMario D’AmicoChunye ZhangMario EstévezChun-Yung HuangMário José Baptista FrancoCinzia BerteaMario MalacarneCinzia FerrarisMario MarchettiClaire C. Berton-CarabinMario SalazarClaire D. MunialoMario ŠčetarClaire Darizu MunialoMario Vincenzo RussoClaire DUFOURMariola KozłowskaClaire Sulmont‐RosséMariolina GullìClara CicatielloMarios G. KostakisClara FaresMarios MataragasClara GrossoMarisa Del RíoClara SousaMarisa PalazzoClara TalensMarisol VillalvaClarisse NobreMaristella GussoniClaudia CampanaleMariusz FlorekClaudia DesiderioMariusz HartmanClaudia FoersterMariusz TichoniukClaudia GenoveseMariusz Tomasz DziadasClaudia GiordanoMariza LandgrafClaudia Gonzalez ViejoMark A. BerhowClaudia GuldimannMark A. SmithClaudia Maria BalzarettiMark EtzelClaudia Muhle-GollMark SolomonClaudio BelliaMarko JukicClaudio C. OliveiraMarko ObranovićClaudio CassinoMarlies Hörmann-WallnerClifford HallMarta BertolinoClizia VillanoMarta ChmielColette BESOMBESMarta CiecierskaColin G. ProsserMarta CitelliConcepción Pérez-LamelaMarta Ferreiro-GonzálezConcetta NazzaroMarta GallegoConor O'HalloranMarta H. F. HenriquesConrad O. PereraMarta HerreraConrad PereraMarta LaranjoConstantinos K. ZacharisMarta Linares-ManriqueConstantinos V. NikiforidisMarta López AlonsoCorinne Pau-RoblotMarta Mesías GarcíaCorrado CostaMarta NevesCorrado RizziMarta OlechCosima D. CalvanoMarta PasławskaCosmas NathanailidesMarta SajdakowskaCostanza CeccantiMarta Tomczyńska-MlekoCraig HedbergMartha Graciela Ruiz-GutiérrezCretenet MarinaMartin GandCristian BonviciniMartin Krøyer RasmussenCristian RamírezMartin KulmaCristian TreleaMartin PtacekCristian VillaMartin SteupCristiana NunesMartina BednářováCristiana TorresMartina BituhCristina BotinesteanMartina GastlCristina Cejudo BastanteMartina JakovljevicCristina Delgado-AndradeMartina LindnerCristina González‐DíazMartina LoiCristina Juan GarcíaMartina LudewigCristina MaccalliniMartina Srajer GajdosikCristina Marcucci MariaMartins SabovicsCristina Martínez-VillaluengaMarwa R. AliCristina MellinasMary Ellen CamireCristina MinnelliMary Katherine HoyCristina Miranda GuedesMary McCarthyCristina Molina RosellMaryam GharachorlooCristina Perez-SantaescolasticaMaryam Nikbakht NasrabadiCristina ProserpioMaryia MishynaCristina RochaMarzena Danowska-OziewiczCristina SaraivaMarzena Jeżewska-ZychowiczCristina SgherriMarzena TomaszewskaCristina SoaresMarzena Włodarczyk-StasiakCristina TruzziMarzena ZajacCristino Alberto Gómez LucianoMarzena ZającCsilla BenedekMarzia IngrassiaCustódia GagoMarzia PezzolatoCynthia BlantonMarzia VasarriCyril ColasMarzieh HosseininezhadDa ChenMarzieh KamankeshDae-Hee LeeMaša BuljacDag EkebergMasaharu KurodaDagmara ZłotkowskaMasaki HondaDaiki MurayamaMasanori AritaDaisuke KobayashiMasashi AndoDalibor ŠatínskýMasashi InafukuDaliborka Koceva KomlenićMasashi UemaDalma RadványiMasayuki OkudaDal-Woong ChoiMasoumeh BejaeiDamiana ScuteriMassimiliano D'ImperioDan RamdathMassimiliano GasparriniDana MiddendorfMassimiliano OrsiniĐani BenčićMassimiliano RinaldiDani DordevicMassimo CalìDaniel Alexandre NeuwaldMassimo CarossaDaniel BlockMassimo CastellariDaniel BuresMassimo D'ArchivioDaniel CaballeroMassimo De MarchiDaniel Franco RuizMassimo FaustiniDaniel Henrique BandoniMassimo IorizzoDaniel I. OnwudeMassimo MozzonDaniel Jacobo-VelazquezMatej PospiechDaniel MorseMateus Henrique PetrarcaDaniel VecchiatoMathew Hoani CummingDaniel WillettMathias HutzlerDaniel WunderlinMatteo Del NobileDaniela BeckerMatteo MasottiDaniela BeghelliMatteo PeriniDaniela BraconiMatteo ScampicchioDaniela Caetano GonçalvesMatteo TonezzerDaniela CampanielloMatthaios P. KavvalakisDaniela CovinoMatthew B. McSweeneyDaniela De Araujo Viana MarquesMatthew ClarkDaniela GwiazdowskaMatthew J Powell-PalmDaniela HanganuMatthew JoynerDaniela HorvatMatthew S. RecsetarDaniela ImperioMatthias RädleDaniela Manila BianchiMatthias WiggenhauserDaniela MartiniMattia Di NunzioDaniela PacificoMattia RapaDaniela RagoMaud LangtonDaniela RussoMaura Téllez-TéllezDaniela Sánchez AldanaMaurice O’SullivanDaniele BassiMaurizio AcetoDaniele CarulloMaurizio CianiDaniele NaviglioMaurizio RuzziDanielle GreenbergMaximilian Joseph BrolDanijel KarolyiMayura VeeranaDanijela AšpergerMd. Abdul HannanDanijela Bursac KovacevicMd. Meftaul IslamDanka BukvickiMd. Moshfekus Saleh-e-InDanko MiloševićMd. Rajibur Rahaman KhanDanny CliceriMduduzi Paul MokoenaDanuta JaworskaMee Ree KimDanuta Kołożyn-KrajewskaMee-Ra RhyuDanuta PentakMehboob SheikhDanyang YingMe-Hea ParkDaria KolotovaMei-Lin TsaiDario FrisoMelinda FogarasiDario KremerMelissa BakerDario PaoloMena RitotaDarius CernauskasMeng-I (Marie) KuoDariusz DzikiMercè BalcellsDariusz GóralMercè GranadosDariusz KokoszyńskiMercedes CarecheDariusz KucharczykMercedes M. PedrosaDariusz M. StasiakMercedes Vazquez EspinosaDarko PreinerMetka HudinaDarko VelićMeysam SarsharDarna L. DufourMia KurekDasha MihaylovaMian RiazDashnor NebijaMicha HoracekDavid BealeMichael AntoniouDavid ElsweilerMichael AppellDavid GallarMichael B. PapahDavid HopkinsMichael BariotakisDavid Juárez VarónMichael EskinDavid MoseleyMichael G. KontominasDavid NelsonMichael G. WellerDavid Perez NeiraMichael GaenzleDavid ŠilhaMichael HellwigDavid SykoraMichael JahnckeDavid Talens-PeralesMichael KeusgenDavid ThomasMichael KontominasDavide BarrecaMichael LentzeDavide BertelliMichael MeirDavide De AngelisMichael MurkovicDavide GoriMichael NetzelDavide SlaghenaufiMichael R. ReedDavor ValingerMichael S. SagmeisterDa-Wei HuangMichael SwashDawid MadejMichael T. NickersonDawid MikulskiMichael TilleyDe Keukeleire Denis D.Michael TunickDeana JonesMichael WeichhausDebanjana GhoshMichaela HavrlentováDebojyoti MoulickMichail MichailidisDeepak KumarMichailidis MichailDelphine M. PottMichał BijakDemetris KafourisMichał GondekDenes DlauchyMichal JaniakDenis RoyMichal JurasekDennis CladisMichał MiłekDennis L. SemanMichał ŚwiecaDequan ZhangMicheál O’MahonyDerek StewartMichela GrossoDetlef BartschMichela VerniDeyang XuMichele D'AmbrosioDiana Ansorena ArtiedaMichele DeflorioDiana BehsnilianMichele FacciaDiana De SantisMichele MauriDiana Martín GarcíaMichele NavarraDidar UcuncuogluMichelle Ji Yeon YooDidem AykasMichiaki YamashitaDidier STUERGAMiglė BacevičienėDiego BreviarioMiguel A. Aguilar-GonzálezDiego F. TiradoMiguel A. PrietoDiego PlanetaMiguel Angel Fernández-MuíñoDietrich MädeMiguel Ángel Hurtado OlivaDiletta BalliMiguel Ángel LópezDima Faour-KlingbeilMiguel Ángel PrietoDimitra DimitrellouMiguel Ángel Rodríguez-DelgadoDimitra LantzourakiMiguel Ángel Sogorb SánchezDimitri PoddigheMiguel Carmona-CabelloDimitrina Zheleva-DimitrovaMiguel FariaDimitrios FanourakisMiguel MontoroDimitrios GerasopoulosMiguel Rebollo-HernanzDimitrios KontogiannatosMiguel RibeiroDimitrios SklirosMihaela Badea DoniDina Rešetar MaslovMihaela CotârlețDinesh DeochandMihaela Cristina BaicanDiofanor AcevedoMijat BožovićDiogo Thimoteo Da CunhaMi-jeong KimDirk DannenbergerMike LewisDirk LachenmeierMike SissonsDirk W. LachenmeierMiklós NagyDitte B. HermundMilad HadidiDivine Bup NdeMilan Kumar LalDjenane DjamelMilena IvanovićDjin Gie LiemMilena PetriccioneDolores CorellaMilena RašetaDolores González De LlanoMilena RizzoDolores R. SerranoMilica AćimovićDomenico GabrieleMilna Tudor KalitDomenico MontesanoMin ParkDomenico MorroneMina K. KimDomenico RongaiMina LeeDominic AgyeiMindaugas LiaudanskasDominic NarangMing-Chang WuDominico A Guillén-SánchezMingchih FangDominik KmiecikMing-Kai ChernDominik MierzwaMing-Yu LungDominika GłąbskaMinh HaDominika GuzekMin-jung KoDominika KuligMin-Kyu LeeDominika Średnicka-ToberMinwei XuDominique Hurtaud-PesselMioara BercuDominique Larrea-WachtendorffMircea Adrian OroianDonald C. BeitzMircea VinatoruDonald E. SprattMirco VaccaDonata Tania VerguraMirela KopjarDonatella AlbaneseMirela PlaninićDonatella Degl'innocentiMirella De BellisDonato AngelinoMiriam Ortega-HerasDonato Di VenereMiriam SelaniDong Uk AhnMiriam TufarielloDongheon LeeMiriam ZagoDong-Un LeeMirian PateiroDongyup HahnMiriana DuranteDora MelucciMirjana B. PešićDorota Cais-SokolińskaMirko D. De RossoDorota DerewiakaMirko De RossoDorota GałkowskaMirna Fuka MrkonjičDorota MiśtaMiroslav FišeraDorota Najgebauer-LejkoMiroslav HadnađevDorota NowakMiroslav JůzlDorota SosnowskaMiroslava KačániováDorota Tichaczek-GoskaMirosław AniołDorota WojniczMirosław KarpińskiDorota ZielińskaMirsada SalihovicDorota ŻyżelewiczMiryam Amigo-BenaventDouglas GoffMisook KimDouglas RaynieMitsuru FukudaDo-Yeong KimMizuki TanakaDragan BubaloMladen BrnčićDragan MilićevićMoamen M. ElmassryDubravka NovotniMobashwer AlamDubravka ŠkevinMohamad Mazen HAMOUD-AGHADubravka ŠkrobotMohamed Al-FatimiDubravka Vitali ČepoMohamed Ali Abdel-RahmanDya Fita DibweMohamed Aymen ChaouchE. Radziejewska-KubzdelaMohamed El-MeseryEderlan S. FerreiraMohamed F. AbdallahEdgar CambazaMohamed Fathi AbdallahEdgar Chambers IVMohamed GhoulEdgar Delgado-EckertMohamed KoubaaEdit CsapoMohamed MahfouzEdoardo LongoMohammad Ali HesarinejadEdson SilvaMohammad Bagher HassanpouraghdamEduarda Gomes-NevesMohammad Jamshed SiddiquiEduardo CostaMohammad Khairul AlamEduardo DellacassaMohammed El-MagdEduardo Domínguez MedinaMohammed GagaouaEduardo GuzmánMohammed SabbahEduardo Hernández YáñezMohd. Zaheen HassanEduardo SantellanoMojca Čakić SemenčićEdurne Simón MagroMojtaba KavianiEdyta Kowalczuk-VasilevMojtaba ShafieeEdyta MolikMomoko KitaokaEdyta Nalewajko-SieliwoniukMónica BuenoEffie HatzdimitriouMonica FloresEila P. JärvenpääMonica GalloEirini AivazidouMonica LaureatiEirini AnastasakiMonica Rosa LoizzoEleftherios AlissandrakisMonica SandenElena ArenaMonica TarceaElena BancalariMonica TrifElena BozzettaMónica Venegas-CalerónElena CanellasMonika BarbaricElena CunicoMonika BorkowskaElena DreassiMonika CotonElena FranciosiMonika DąbrowskaElena PiniMonika DymarskaElena PoverenovMonika GarbowskaElena Sánchez LópezMonika GąseckaElena Sofia IngugliaMonika GibisElena ViganòMonika JanowiczElena-Alexandra AlexaMonika KaraśEleni KasapidouMonika Kordowska-WiaterEleni MalissiovaMonika KosmalaEleni TsantiliMonika Marcinkowska-LesiakEleonora CariniMonika PischetsriederElia RanzatoMonika PrzeorEliana AlvesMonika SujkaEliana PereiraMonika TrzaskowskaElif Inan-ErogluMonika TrząskowskaElisa AleMonika WójcikElisa De ArcangelisMonique LacroixElisa GiampietriMontserrat DuenasElisa MarracciniMontserrat Mor-MurElisa RobottiMootaz SalmanElisabet Navarro-TapiaMorena GabrieleElisabet Segredo-MoralesMorten SivertsvikElisabete CostaMorteza HaghiriElisabeth GuichardMoshe RosenbergElisabetta BonerbaMoshi GesoElisabetta BraviMoslehpour MassoudElisabetta MurruMost Tahera NazninElisabetta SalimeiMottin CamilaElisabetta SavelliMoyu TaniguchiElisabetta SieniMozaniel OliveiraElisabetta TotiMuhammad Bilal SadiqElisete CorreiaMuhammad Faisal ManzoorElisha Otieno GogoMuhammad KhanEliza KostyraMuhammad Nadeem HassanElizabeth MansfieldMuhammad Shahid Riaz RajokaElizabeth NeumannMuhibUr RahmanElizabeth TomasinoMulusew FikereElizabeth ZandstraMuriel GuyardElke AlbrechtMurugesan ChandrasekaranElke UeberhamMusarat AminaElliot S. FriedmanMyeong-Hyeon WangElpidius J. RukambileNaceur HaouetElsa Díaz-MontesNadeesha GunaratneElsa F. VieiraNadia BadolatiElsa M. GonçalvesNadica Maltar-StrmečkiElsa Margarida GonçalvesNadine MoubayedElsa VieiraNae-Cherng YangElsje PieterseNagendra Kumar KaushikElvis ChuaNa-Hyung KimElżbieta Goryńska-GoldmannNaiara Fernandez HernandezElżbieta Hać-SzymańczukNakako KatsunoElzbieta KlewickaNam Hoon KimElżbieta Łodyga-ChruścińskaNaoki MoritaElżbieta Wójcik-GrontNasreddine BenbettaiebEman M. OthmanNatalia CasadoEmanuela Calcio-GaudinoNatalia DrabińskaEmanuela CamilliNatalia Garcia-GonzalezEmanuela ZanardiNatalia JatkowskaEmanuele BoselliNatalia KordulewskaEmanuele CerrutoNatalia ManousiEmidio ScarpelliniNatalia MartinsEmilia AlvesNatalia VilaEmilia FornalNatalija PolovicEmilia Garcia-MorunoNatalio Antonio García HonduvillaEmilia Janiszewska-TurakNataliya P. BgatovaEmiliano José QuintoNatasa KalogiouriEmilie DestandauNataša MikulecEmilija FriganovićNathalie ConnilEmilio CervantesNathalie IofridaEmmanouil KokkinakisNathalie VionnetEmmanouil PapaioannouNathan M. D’CunhaEmmanuel Fortunato GulinoNathaniel J. S. AshbyEmmanuela MagriplisNawaf Abu-KhalafEncarna Gomez PlazaNayan Ranjan SinghaEncarna Gómez-PlazaNazim SekerogluEnoch Owusu-SekyereNazimah HamidEnrico CiniNeal BarnardEnrico Vito PerrinoNeđeljko KarabasilEri Ogiso-TanakaNeelima MahatoEric BadiaNeha RathiEric BourillotNeil D. DanielsonEric Keven SilvaNeil DanielsonErica TirloniNeith PachecoErica VareseNela FilimonErich LeitnerNelson Pérez GuerraErick Cesar VidanaNemanja TeslicErick Paul Gutiérrez-GrijalvaNemanja TeslićErik KuhnNerdy NerdyErik M. PilgrimNeringa RasiukevičiūtėErika BujnaNermeen Adel ElkasabgyErin RosskopfNéstor De La VisitaciónErmes Lo PiccoloNéstor Gutiérrez-MéndezErnesto SotoNevijo ZdolecErnst WolteringNewton N. J. YauErpeng WangNiamh HarbourneErrol HassanNicola A. UccellaEsben EllerNicola CaporasoEssam HebishyNicola CiceroEstefania Muñoz AtienzaNicola FrancescaEsther AriasNicola GasparreEsther Mayer-MiebachNicola LandiEsther Pérez-CarrilloNicola SimoneEstrella Espada-BellidoNicolai PanikovEugenia BezirtzoglouNicolas BergerEugenio ApreaNicolas KorsakEugenio DemartiniNicole FarmerEugenio ParenteNicole Roberta GiuggioliEui-Cheol ShinNicole WenteEulogio CastroNicoleta-Aurelia ChiraEun Ha ChoiNicoletta FaraoneEunhee ChungNicoletta GuerrieriEunju ParkNicoletta MurruEunmi KohNidal JaradatEva CunhaNidhish FrancisEva DortaNigel DaviesEva García-MartínezNiki AlexiEva M. Santos LópezNiki C. MaragouEva MrkvicovaNikola ČobanovićEva SamkováNikola MajorEva TsaousidouNikolai KuhnertEvandro Leite De SouzaNikolaos KopsahelisEvangelia LivaniouNikolaos NikoloudakisEvangelos ZoidisNikolaos SolomakosEvdokia K. OikonomouNikolina Kelava UgarkovićEvgenia MitsouNikolina NovakovEvgenia ShishkovaNilesh NirmalEwa Czarniecka-SkubinaNima HematyarEwa DomianNino TerjungEwa GondekNiny Z. RaoEwa JakubczykNirit BernsteinEwa LangeNirmala KandadaiEwa MajewskaNiroz Abu-Saleh ZoabiEwa Ostrowska-LigȩzaNishikant WaseEwa Ostrowska-LigęzaNives Marušić RadovčićEwa Pecka-KiełbNobuyuki SakaiEwa PiątkowskaNoé OntiverosEwa RembialkowskaNoelia Caballero-CaseroEwa Sadowska-KrępaNoemi BaldinoEwa SzpyrkaNoemi ProiettiEwa TomaszewskaNolwenn TermeEwa WasilewskaNor QhairulEwa ZdybelNóra AdányiEwelina BasiakNorbert RaakEwelina HallmannNorio SogawaEwelina KowalczykNoura DosokyEwelina PatyraNuno Bartolomeu AlvarengaEwelina Pogorzelska-NowickaNuno CrespoEwelina ZielińskaNuno LunetEyal SeroussiNuno RodriguesEzequiel CoscuetaNunzia CiccoF. J. E. AlcaideNuria TousF. Xavier MedinaOana Emilia ConstantinFabian SzepanowskiObyedul Kalam AzadFabiano A. N. FernandesOctavio Dublan-GarcíaFabiano CapparucciOdile Francesca RestainoFabio BrambillaOgueri NwaiwuFabio Di NardoOlaia Martínez GonzálezFabio FavatiOlga EscuderoFabio Gaetano SanteramoOlga Monago-MarañaFabio LicciardelloOlga OrynyczFabio MasottiOlga Padilla-ZakourFabio SalafiaOliver A. H. JonesFabio SciubbaOliver MeixnerFabio VerneauOlivera DjuragicFabrice BerrueOlivera GalovićFabrice TouzainOlívia R. PereiraFabrizia TittarelliOlivier DanglesFabrizio CapoccioniOlivier FirmesseFadi M. AramouniOlivier RuéFadia Victoria Cervantes DomínguezOlman Gómez-EspinozaFady MoharebOnofrio CoronaFanbin KongOran KwonFangcong DongOrsolina PetilloFanny TsironiOscar Daniel Rangel HuertaFarayde Matta FakhouriOscar López CamposFaraz MuneerOscar Martínez-AlvarezFareyde Matta FakhouriÓscar Martínez-ÁlvarezFarid ChematOscar NunezFarnaz Ghazi-NezamiOskar SzczepaniakFatemeh Mohammadi-NasrabadiOsmar Antonio Jaramillo-MoralesFatemeh NejatiOthmane MerahFátima Alves MillerOvergaauw PaulFatima El KhalloufiOwen Griffith JonesFatima Lambarraa-LehnhardtOzgur TarhanFaustino Merchán SorioPablo A. HenríquezFausto TintiPablo FernándezFederica BlandoPablo J. ArauzoFederica BonelloPablo MirallesFederica Di CesarePajor FerencFederica IanniPaloma ManzanaresFederica SabuziPanagiota KatikouFederica TaddeiPanagiota TsafrakidouFederica TinelloPanagiotis GeorgiadisFederica TonoloPanagiotis KandylisFederico BiagiPanagiotis SimitzisFederico CapuanoPankaj B. PathareFederico CarosioPanmanas SirisomboonFederico CasanovaPantelis NatskoulisFederico Di RitaPaola BadinoFederico StiloPaola CeteraFedor LisetskiiPaola MontoroFei HePaola StiusoFeifei TaoPaola TedeschiFeilong LiPaolo ContiFelipe Reinoso CarvalhoPaolo D'InceccoFelix ClanchyPaolo GabrielliFelix Jimenez-RondanPaolo LucciFerdinando BrancaPaolo MarianiFereidoon ShahidiPaolo MeneguzzoFernanda CosmePaolo MerellaFernanda F. G. DiasPaolo PolidoriFernanda GalganoPaolo RomaFernanda Maria VaninPaolo S. CalabròFernanda PeyronelPaolo TrerotoliFernanda VilarinhoPaolo Usai SattaFernández-Ocaña AnaPaolo ZambonelliFernando PablosPapadopoulou OlgaFernando Ramos-EscuderoParimalan RanganFideline Laure Tchuenbou-MagaiaParis D. N. SvoronosFilip DujmićParise AdadiFilipa MonteiroParthasarathi SubramanianFilippo GiarratanaPascal SchlichFilippo RossiPascal Wegmann-HerrFilomena CorboPaschalitsa TryfinopoulouFilomena L. DuartePasquale CatzedduFilomena Monica VellaPasquale FerrantiFilomena NazzaroPasquale GiungatoFiorella SinesioPasquale RussoFioretta AsaroPatricia A.B. RamosFlavio BocciaPatrícia BatistaFlavio TidonaPatricia CarloniFlora Valeria RomeoPatricia CombarrosFlorentina-Daniela MunteanuPatricia Duque EstradaFlorian FischerPatricia García-HerreraFlorian FreimoserPatricia García-MoraFodor MariettaPatricia Gómez MoralesFohad HusainPatricia HarveyForough JahandidehPatricia J. HarveyFortunato Palma EspositoPatricia Rayas-durteFoteini F. ParlapaniPatricia Reboredo-RodríguezFoteini PavliPatricia RegalFouad Ali Abdullah AbdullahPatricia VázquezFranca AngerosaPatricio Orellana-PalmaFranca FinocchiaroPatrick BradshawFrancesc Fusté-FornéPatrick RollinFrancesc SepulcrePatrick SchenkFrancesca ArfusoPatrik BurgFrancesca BlasiPatrizia FavaFrancesca ContePatrizia FronteraFrancesca CuomoPatrizia RisoFrancesca De FilippisPatrizia RomanoFrancesca MalvanoPatrizia ZavattariFrancesca MasinoPatrycja MakośFrancesca MeliniPaucean AdrianaFrancesca NocentePaul A. BreretonFrancesca PedonesePaul B. SiegelFrancesca R. BertaniPaul BakerFrancesca RaganatiPaul ShipleyFrancesca RiganoPaula A. OliveiraFrancesca RiuzziPaula Alexandra A.B. LopesFrancesca SparvoliPaula BarbosaFrancesca ValerioPaula BorrajoFrancesca VenturiPaula C. CastilhoFrancesco BarrecaPaula CorreiaFrancesco BimboPaula Garcia-OliveiraFrancesco BuonocorePaula LeslieFrancesco CacciolaPaula Mapelli-BrahmFrancesco CaponioPaula PereiraFrancesco ChiesaPaul-Alexandru PopescuFrancesco CiminoPaule V JosephFrancesco CorriasPauliina PalonenFrancesco DesogusPaulina BierzuńskaFrancesco EspositoPaulina KęskaFrancesco FancelloPauline AntonFrancesco Garbati PegnaPaulo De Souza Costa SobrinhoFrancesco GenovesePaulo E. S. MunekataFrancesco GeobaldoPaulo MunekataFrancesco GriecoPavel DivisFrancesco LongobardiPavel DostalekFrancesco LopezPavel KloučekFrancesco MartelliPavel KopelFrancesco MeneguzzoPavel NevrklaFrancesco MollicaPavel SomavatFrancesco Paolo FanizziPavlos TsouvaltzisFrancesco PomilioPavol GenzorFrancesco SavoraniPawel BrylaFrancesco SianoPaweł GlibowskiFrancesco VisioliPawel KafarskiFrancine M. GiottoPaweł KafarskiFrancis ButlerPaweł KrzyżekFrancis MuchaambaPaweł PtaszekFrancisca HernándezPaweł SobczakFrancisca Isabel BravoPayam MehrshahiFrancisca RodriguesPayam ZarrintajFrancisco A.P. CamposPedro A. BurdaspalFrancisco Areal BorregoPedro A. R. FernandesFrancisco C IbañezPedro AraujoFrancisco Entrena-DuranPedro González-RedondoFrancisco Humberto Xavier-JuniorPedro MenaFrancisco J. BarbaPedro Palos-SánchezFrancisco J. Rodriguez-PulidoPedro Rodríguez-LópezFrancisco J. Salafranca LázaroPedro SilvaFrancisco Javier Alarcón LópezPenelope Perkins-VeazieFrancisco Javier Leyva-JimenezPenghao WangFrancisco Javier MesíasPengyi ZhaoFrancisco José OstosPere PuigdomènechFrancisco Jose Sarabia SanchezPerea Muñoz José ManuelFranco CervellatiPeriaswamy Sivagnanam SaravanaFranco J. PagottoPete WildeFranco Van De VeldePeter Christopher KonturekFrangipane Maria TeresaPéter FodorFrank ThieleckePeter J. T. DekkerFrank WellePeter J. WildeFranklin Brian Apea-BahPeter KoehlerFranks Kamgang NzekouePeter LillfordFranz BucarPeter PaulsenFred KhachikPeter R. LaityFrédérique PasqualiPeter TimmermanFriedrich-Karl LückePeter W. ThulstrupFufeng LiuPetr MarsikFuhito HojoPetra FörstG. K. SivaramanPetra Hlásná ČepkováGabriela ChiciudeanPetra TerpincGabriela GrigioniPetri KilpeläinenGabriela Lopez-VelascoPetros KatapodisGabriela RapeanuPetros TarantilisGabriela Vollet MarsonPhil BassGabriele CarulloPhil BremerGabriele Di MarcoPhilip PrinzGabriele DomenicoPhilippe CayotGabriele KrczalPhilippe LebaillyGabriele RocchettiPhillip E. StrydomGabriele ScozzafavaPhotini MylonaGabriella GiovanelliPiedad GañánGabriella SavianoPiera Di MarzioGabriella Ten HavePiero BruschiGaelle RoudautPiero FranceschiGaetano CammilleriPierre PétriacqGaetano ChinniciPierre Sylvain MiradeGaetano MartinoPierre-Alexandre ChâteauGajdoš Kljusurić JasenkaPierre-Yves PontalierGal KreitmanPietro PulinaGalia ZamaratskaiaPietro RocculiGanesan NarsimhanPin-Der DuhGap Don KimPiotr BórawskiGap-Don KimPiotr DomaradzkiGar Yee KohPiotr GolińskiGarima KulshreshthaPiotr JaniszewskiGarmt DijksterhuisPiotr KulawikGarth TarrPiotr KuśGary D. RaysonPiotr MinkiewiczGary StonerPiotr SkałeckiGary WilliamsonPiotr SugierGaryfallia KapravelouPiotr SzwedaGaspar Ros BerruezoPiotr SzymańskiGaud Dervilly-PinelPiotr WieczorekGavino SannaPiotr ZarzyckiGayathri BalakrishnanPo-Hsien LiGeir Sogn-GrundvagPoonam SharmaGemma Gutiérrez CervellóPooria PasbakhshGemma ReigPopa Mona ElenaGemma VillorbinaPorfirio Gutierrez MartinezGeneviève Gésan-GuiziouPoul Erik JensenGengjun ChenPo-Wen ChenGeng-Ruei ChangPo-Yuan ChiangGeorg SurberPrabin LamichhaneGeorge AngelovPramod V. MahajanGeorge BoskouPredrag PutnikGeorge KatsarosProdromos SkenderidisGeorge LazaroiuProspero Di PierroGeorge M. SavvaPrzemysław KosobuckiGeorge N. HotosPrzemyslaw Lukasz KowalczewskiGeorge SymeonPuja KumariGeorge VlontzosPurificación García-SegoviaGeorgi KostovQian YangGeorgia MandilaraQinchun RaoGeorgia ZoumpopoulouQiuchen DongGeorgia-Eirini DeligiannidouQiu-Jin ZhuGeorgiana CodinaQuang D. NguyenGeorgios BanilasQuoc Cuong NguyenGeorgios BlekasR.A. ShallikerGeorgios DiamantopoulosRadka HulánkováGeorgios TsaniklidisRadmila PavlovicGeorgios XanthopoulosRadoslaw DrozdGerald SchwerdtRadosław KowalskiGerard DumancasRadosław SpychajGérard Liger-BelairRadoslaw WiniczenkoGérard LizardRadu C. RacovitaGerardo Alvarez-RiveraRafael GavaraGerardo CentoducatiRafael Guillén-BejaranoGerardo NavaRafael Ravina-RipollGerd KnuppRafael ZárateGergely SzolnokiRafał MalinowskiGerhard GebauerRafal OgorekGermano MucchettiRafał WołosiakGerold BesserRaffaela BiesuzGerson Lopes TeixeiraRaffaele CampoGerui RenRaffaele RomanoGhanendra GartaulaRaffaella BoggiaGhoson DabaRaffaella MicilloGiacomina BrunettiRaffaella PretiGiacomo SqueoRaghavendra AmachawadiGiacomo ZaraRagita C. PramudyaGiampaolo ColavitaRaimund NagelGianfranco PannellaRaivo KalleGianluca GiubertiRajbir SinghGianluca ViscusiRajesh Chandra MisraGianluigi MaurielloRajib ShawGianna PalmieriRajiv Kumar KarGianni PastoreRajka BozanicGigliola BorgonovoRalf BlankGilberto GiangoliniRamapraba AppannaGilda AielloRamesh Kumar SainiGilles TrystramRamón Arias SánchezGinés B. Martínez–HernándezRamon ArmengolGino CiafardiniRamón CavaGioacchino PappalardoRamón MoreiraGiorgia BorrielloRamona IseppiGiorgia LiguoriRana Muahammad AadilGiorgio CapuaniRana Muhammad AadilGiorgio GiraffaRandy AdjonuGiorgio GrilloRanga Rao AmbatiGiorgio SalutiRaquel AjatesGiorgio SchifaniRaquel Braz Assunção BotelhoGiorgio SmaldoneRaquel GarciaGiovanna EspositoRaquel IbarzGiovanna FiaRaquel OlíasGiovanna GiovinazzoRaquel P. F. GuinéGiovanna LonghiRaquel RialGiovanna SacchiRaquel SanchezGiovanna SperanzaRaquel Sánchez-FernándezGiovanni AgatiRasangi WimalasingheGiovanni AntoniniRathinam RajaGiovanni Antonio LutzuRatmawati MalakaGiovanni BartolomeoRaúl Ferrer-GallegoGiovanni De FrancescoRaul MoralGiovanni D'OrazioRavi PandiselvamGiovanni NieroRay-Yu YangGiovanni PeiraRaziye PourdarbaniGiovanni PreitiRebeca Fernández-CarriónGiovanni RibaudoRebecca MilczarekGiovanni SogariRebecca SchmidtGiraudo AlessandroRegina KratzerGisèle LaPointeRegina WierzejskaGislaine FongaroRegine SchönlechnerGiulia AbateRégine TalonGiulia BianchiReham Hassan MekkyGiulia MarroneReiner JedermannGiulia SecciReinhard EderGiuliana VinciRemedios CastroGiulio MarchesiniRemigiusz BąchorGiuseppa Di BellaRenata Gadzała-KopciuchGiuseppa GenoveseRenata KazimierczakGiuseppe ApreaRenata Markiewicz-ZukowskaGiuseppe BlaiottaRenata NassuGiuseppe CavallaroRenata RabeloGiuseppe CinelliRenata Raina-FultonGiuseppe Di VitaRenata RóżyłoGiuseppe EspositoRenata SõukandGiuseppe ManiaciRenata ZandonadiGiuseppe ManninoRenato B. PereiraGiuseppe MartanoRenato BruniGiuseppe MartinoRene A. de WijkGiuseppe MecaRené BreuchGiuseppe MuratoreRené LafontGiuseppe ZanottiReshma B. NambiarGiuseppe ZeppaRestituto TocmoGiuseppina CaggianoRevoredo-Giha CesarGiuseppina MandalariRewa RaiGiuseppina Marilia TantilloRey David Vargas-SánchezGiusi MacalusoReza TahergorabiGiverson MupambiRicard BoquéGleidson Giordano Pinto De CarvalhoRicard BouGloria Jiménez-MarínRicardo Costa CalhelhaGloria LoboRicardo EgiptoGloria María Molina SalinasRicardo González-RezaGloria Márquez-RuizRicardo M. F. Da CostaGniewko NiedbałaRicardo MeloGolfo MoatsouRicardo N. PereiraGonçalo RodriguesRicardo PáscoaGonzalo MirandaRiccardo IentileGopinadhan PaliyathRiccardo MassantiniGopinathan H. MeletharayilRiccardo TestaGopinathan JanarthananRicha SalwanGöran EricssonRichard J FitzGeraldGoran KusecRichard MithenGordana Blagojevic ZagoracRie MukaiGordana ĆetkovićRie TomizawaGordana ŠimićRifat Ullah KhanGordon C. TuckerRita Carneiro AlvesGordon SellingRita CelanoGoutam MondalRita PombinhoGraeme HeronRob KingsleyGrahame MackenzieRobert BrinsonGrazia CasielloRobert BuchananGrazia QueroRobert DambergsGraziele Grossi Bovi KaratayRobert G. ElkinGrażyna AdamusRobert MaddockGrażyna BudrynRobert RusinekGrażyna Cacak-PietrzakRobert SochaGrażyna KowalskaRobert TylerGrazyna LewandowiczRobert W. OrttungGrażyna LewandowiczRoberta CampardelliGrazyna NeunertRoberta CazzolaGrażyna ZgórkaRoberta CongestriGreta AdamczykRoberta D’AurelioGrethe HyldigRoberta FoligniGretty VillenaRoberta GalariniGrigoris AmoutziasRoberta MerlantiGro Haarklou MathisenRoberta MoruzzoGrzegorz BartoszRoberta ParenteGrzegorz WęgrzynRoberta PreteGrzegorz ZagułaRoberta TolveGuadalupe Molina TorresRoberto AneddaGuan-James WuRoberto Castro-MuñozGuan-Wen ChenRoberto CiccorittiGudrun OlafsdottirRoberto FabianiGuerrino MacoriRoberto GiovannoniGuido Di MartinoRoberto Martínez BeamonteGuido DurianRoberto MoscettiGuido R. LopesRoberto RomanielloGuillaume L. ErnyRoberto Romero-GonzálezGuillermo CebriánRoberto ScicaliGuillermo Garcia-GarciaRoberto StellaGuillermo RipollRocchina PietrafesaGUILLERMO RODRÍGUEZRocío CasqueteGulam RabbaniRodica DinicăGulnihal OzbayRodrigo A. IbáñezGulzar Ahmad NayikRodrigo Cardoso De OliveiraGuoqing WangRodrigo GarcíaGuoxiang JiangRodrigo MelgosaGuozhou LiaoRogelio Sánchez-VegaGurunathan KandeepanRohit P. DugarGustavo AmoresRohtraud PichnerGustavo Cordero-BuesoRoland KlassenGustavo Di LalloRolf D. JoergerGustavo Fidel Gutiérrez-LópezRomain EspinosaGustavo Nakamura Alves VieiraRomain KapelGustavo PimentelRoman PavelaGüzin KabanRomdhane KarouiGyeongsik OkRomina Alina MarcGyung-Ah WieRommy DíazGyungsoon ParkRonan LordanHack-Yeon KimRon-Hendrik HechelmannHadi ParastarRonit MandalHai-Jin MouRonivaldo Rodrigues Da SilvaHailei WeiRos GleadowHamed BostanRosa Di SanzoHamid B. GhoddusiRosa DireitoHamid PakmaneshRosa Isela Corona-GonzálezHammad BadarRosa Maria De Sá PerestreloHana BuchtováRosa Maria FanelliHang QiRosa Maria Garcia-GimenoHan-Joo MaengRosa María Martínez-EspinosaHanna DudekRosa PalmeriHanna KowalskaRosa PeñalverHanna LewandowskaRosa PerestreloHanna M. KoivulaRosa PilolliHanna MojskaRosa TundisHanna SzaeferRosalia Gracia-TorresHanne Christine BertramRosalind DalefieldHanne Kristine SivertsenRosana RibićHan-Seok SeoRosaria CozzolinoHan-Shen ChenRosaria ViscecchiaHao ChenRosario LinaceroHao Ping ChenRosario MartinsHaotong ChenRosario RamirezHari Prasad DevkotaRosario Sánchez GómezHarley D. DickinsonRose MartinHary L. RazafindralamboRosemary Aparecida De CarvalhoHasitha PriyashanthaRosita RussoHayat BenkhelifaRoss GrantHayati SamsudinRossana RoilaHeather ChannonRossana SidariHeather L. BruceRossella CaporizziHeather MacalisterRossella Di MonacoHefei ZhaoRossella TozziHeikki AisalaRothman KamHeikki VapaataloRóża Biegańska-MarecikHeinar SchmidtRozenn RavallecHekap KimRubén AgregánHélder OliveiraRubén Cebrián CastilloHelen McCarthyRubén Del Barrio-GalánHelen OnyeakaRuben DominguezHelena SilvaRuchir PriyadarshiHelene N. ChinivasagamRudy Caparros MegidoHelle Nygaard LærkeRui CostaHemant Kumar DaimaRui LiuHendryk CzechRui M. S. CruzHenryk H. JeleńRui M. V. AbreuHenryk JeleńRuilong ShengHerbert L. MeiselmanRune RødbottenHerbert Ryan MariniRunze YuHerlinda Soto ValdezRupesh Kumar SinghHerminia Vergara PérezRuslan KubantHidayat HussainRussell McKeithHidefumi YoshiiRusso GiuseppeHidehiko HirakawaRuta GaloburdaHideki KANDARuth Ben-ArieHidemasa IzumiyaRuud J. B. PetersHilda E. GhadiehRyota HosomiHilton DeethRyota MabuchiHiroaki KitazawaRyszard AmarowiczHiroshi HaraRyszard CierpiszewskiHiroshi KitagakiRyszard KuligHiroshi MatsufujiSaber AmiriHiroshi OgawaraSabina GalusHiroyuki HondaSabina GórskaHiroyuki KataokaSabine HarrisonHiroyuki NakamotoSabrina MoretHiroyuki YanoSadia AfrinHitoshi ShirakawaSaeed MozaffariHong Ngoc Thuy PhamSaeid AsadpourHongbing FanSaid GharbyHongbing LiuSajad Ahmad SofiHong-Jhang ChenSajad SabziHongshun YangSajid AliHong-Ting Victor LinSalih KarasuHongying DuSally EldeghaidyHoonsoo LeeSalvador RosellóHosam O. ElansarySalvatore Antonino RaccuiaHossein HaghighiSalvatore BarberaHouda BerradaSalvatore BarrecaHrvoje PavlovićSamantha J. NoelHsi-Mei LaiSamantha M. GromekHsin-Bai YinSameer Al-BatainehHsin-Chih LaiSami I. AliHsin-I ChiangSamir KalitHsin-Tang LinSamir SmettiHsu Yang LinSamo SmrkeHsun-Shuo ChangSampat GhoshHsu-Sheng YuSamuel Perez-VegaHuaiwen YangSandeep JagtapHualu ZhouSander WuytsHubert AntolakSándor GondaHubert SzczerbaSandra HelinckHubertus HimmerichSandra HillHugo Cunha Da SilvaSandra OsésHugo Espinosa AndrewsSandra PedisićHuijuan ZhengSandra PetricevicHuiying ZhangSandra PintoHujun XieSandra RivasHung-Ju LiaoSandra Sofia Quinteiro RodriguesHung-Pin TuSandra ZavadlavHushton BlockSandrayee BrahmaHuzefa RajaSandrine GuillouHwa-Jin LeeSandrine Monnery-PatrisHye Won KimSang Guen KimHyeyoung LeeSang Hyeon LeeHyunbin JoSangbin LimHyun-Dong PaikSangdoo AhnHyun-Wook ChoiSang-Han LeeHyun-Wook KimSang-Ho YooIain A. BrownleeSangita GangulyIan TetlowSangkyu JungIbrahim KhalifaSangmi LeeIda OreficeSanjeet MehariyaIdaira Pacheco-FernándezSanna ManuelaIfeanyi NwachukwuSanna MeriläinenIga RybickaSantiago AubourgIgino AndrighettoSantiago Hurtado-BermúdezIgnacio BejaranoSantoshi MuppalaIgnacio FernándezSara BarbieriIgor JerkovićSara BenedéIgor ŁoniewskiSara BernardoIgor LukićSara CanasIgor Piotr TurkiewiczSara ErasmusIgor SepelevsSara MoradiIkram BelghitSara PrimavillaIlandarage Menu Neelaka MolagodaSara SilvaIlaria De PasqualeSarah AzinheiroIlaria PelusoSarah CurròIlda CaldeiraSaraswoti NeupaneIliada LappaSarka HorackovaIlias GiannenasSathiyaseelan AnbazhaganIlja Gasan Osojnik ČrnivecSathyanarayanan Sevilimedu VeeravalliIlona OlędzkaSatish Kumar RajasekharanIlona SadokScott FrostIman ZareiScott LafontaineImen HamedScott M. HoltImmacolata Cristina NettoreSeamus O'MahonyImmacolata FaraoneSean David GallaherImmaculada PascualSean O’KeefeImre BoldizsárSebastian GranicaImre HolbSebastian KotInaki ElioSebastian StępieńInes KüsterSebastiano GarroniInês N. RamosSeiichi MatsugoIngars ReinholdsSeinen ChowIngeborg KlymiukSenaka RanadheeraIngela MarklinderSenem KamilogluInge-Willem NoordergraafSenorpe Asem-HiablieIngrid Mayanin Rodriguez BuenfilSeojin ChungIngunn BergetSeok Shin TanIngunn SamdalSeon-Yong JeongIngvar SundhSerena LimaIngvar SvanbergSerena MandolesiInmaculada FrancoSerena NiroInmaculada GómezSerena SantonicolaInmyoung ParkSerge HercbergInsin KimSergi MaicasIn-Soo KongSergio I. Martínez-MonteagudoIoanna MantzouraniSergio MonteagudoIoanna NtaikouSergio Pérez-BurilloIoanna S. KosmaSergio RivaroliIoannis BakoyiannisSerkan Serkan SelliIoannis GiavasisSerrano SaludIoannis MourtzinosSetu Bazie TageleIoannis N. PasiasSeung Hee LeeIoannis S. BoziarisSeung-Joo LeeIoannis ZabetakisŞeyda BostancıIoannis Ν. XyniasSghaier ChrikiIolanda AltomonteShafi SahibzadaIonel BostanShaghef EjazIonica OncioiuShahzad IqbalIppolito CameleShanmugam HemaiswaryaIrena RotermanSharmila FagooneeIrena ZuntarSharmin HasanIrene CaffaSharon PuleoIrene (Eirini) KamenidouShashi Kant BhatiaIrene FalasconiShauna C. HenleyIrene KamenidouSheila SandersIrene PanderiSheryl BarringerIrene RomeroSheta M. ShetaIreneusz KapustaSheu FuuIreneusz OchmianShigeaki UenoIrfan A. RatherShih-Tse WangIrina TorresShih-Yi HuangIrineu BatistaShin-ichi KayanoIrini DoytchinovaShin-Joung RhoIrini F. StratiShinsuke NirengiIrmela SarvanShintaro PangIrwin Alencar MenezesShiqi HuangIrwin R. Donis-GonzalezShiva Ram BhandariIsaac ZiporiShokoofeh ShamsiIsabel AndradeShokouh HaddadiIsabel FernandesShpigelman AviIsabel FerreiraShubham SharmaIsabel Maria Nunes De SousaShu-Chen HsiehIsabel NoguesShunji KatoIsabel Odriozola-SerranoShunsuke FurutaniIsabel RevillaShuvra SinghaIsabel RodriguezSiegrid De BaereIsabel Ten-DoménechSiemowit MuszyńskiIsabella D'AntuonoSigrun J. HaugeIsabella EndrizziSigurdur G. BogasonIsabella Pali-SchöllSijing LiIsabella TaglieriSilvana CavellaI-Shiang TzengSilvani VerruckIsidra RecioSilvia BistiIsmail IshamriSilvia CañasIsmail YenerSílvia Castro CoelhoIsrael MuñozSilvia De LamoIsuru WijesekaraSilvia Di GiacomoItamar GonçalvesSilvia FloresItziar ChurrucaSilvia GrassiIulian Zoltan BoboescuSilvia Laura Guzmán-GutiérrezIva Juranović CindrićSilvia MarzocchiIván Adrián García GaliciaSilvia MironeasaIvan Cruz-ChamorroSilvia NovelliIvan LeguerinelSilvia PampanaIvan Luzardo-OcampoSílvia PetronilhoIvan M. SavicSilvia ScaglioniIvana FlanjakSilvia VincenzettiIvana Generalić MekinićSilvina Cecilia AndresIvana GiangriecoSimona AltieriIvana GobinSimona FabroniIvana HorvatSimona GrassoIvana LončarevićSimona JancikovaIvana MarováSimona Lucia BavaroIvana Savic GajicSimona Marianna SanzaniIvana Tlak GajgerSimona PiccolellaIvano De NoniSimona PrencipeIvo OliveiraSimone AngeloniIvo Vaz De OliveiraSimone BellucoIvona Djurkin KušecSimone BrogiIvona KožárováSimone CavaleraIwata OzakiSimone VincenziIwona BatykSimonetta FoisIwona KomanieckaSinh Dang-XuanIwona Michalska-PożogaSiroj PokharelIwona RudkowskaSirpa KurppaIwona ŚcibiszSithisarn PongtipIwona StawoskaSlađana DavidovićIwona Wojtasik-KalinowskaSladjana SavatovićIyyakkannu SivanesanSlaven JurićIza F. Peréz-RamírezSławomir KulaIzabela GrzegorczykSmarak BandyopadhyayIzabela MichalakSneh PuniaIziar A. LudwigSofia AgriopoulouJ. B. WilliamsSofia ChaniotiJ. F. Ayala-ZavalaSofia K. DrakopoulouJ. L. Gómez ArizaSofia ReisJacek E NyczSohail QaziJacek GawrońskiSol ZamuzJacek GębickiSolmaz TabtabaeiJacek LewandowiczSolomiia KozachokJacek SłupskiSomchai AmornyotinJacek WójtowskiSomdip DeyJacobo Lopez SeijasSong LiJacqueline J. M. CastenmillerSonia BarrosoJacqueline TakahashiSonia CarabettaJacqui M. McRaeSonia EspostoJadranka FreceSónia GomesJadwiga Stanek-TarkowskaSónia PedroJadwiga TurłoSonia PiacenteJae Sue ChoiSonia Sayago-AyerdiJae Sup ShinSoo-Ki KimJaewoo BaiSophie FoleyJae-Young JeSophie KittlerJagoda O. SzafrańskaSophie LandaudJaime Amaya FarfanSophie TempereJaime AnibalSophie Thetiot-LaurentJaime De Pablo ValencianoSoraya Paz MontelongoJaison JeevanandamSorel Sagu TchewonpiJames BolesSorin MunteanJames Owusu-KwartengSotirios I. EkonomouJan BocianowskiSotirios KaravoltsosJan Mei SoonSoukaïna El-GuendouzJan MieszkowskiSpiros ParamythiotisJan Willem Van Der KampSpyridon PetropoulosJana OlsovskaSrinath PashikantiJana SedlaříkováSrinivas SistlaJana Šic ŽlaburSrinivasan RamalingamJanak K. VidanarachchiStanisław MlekoJane HsuStavros KalogiannisJanice SychStefania SavoiJanosch HellerStefanos LeontopoulosJanusz GołaszewskiStella LignouJara LasoStephan SommerJari E. KarppinenStéphane BalayssacJarmila CelakovskaStephanie K. NishiJaromir LachmanStephen InbrajJaroslaw CzubinskiStephen JonesJaroslaw CzubinskyStyliani MinoudiJarosław KorusStyliani ProtonotariouJaroslaw KowalikSung Keun JungJaroslawa RutkowskaSunghyup Sean HyunJasenka Gajdoš KljusurićSung-Yong HongJaslyn LeeSun-Young LeeJasmina LukinacSusan LurieJasmina Lukinac ČačićSusan SanchezJasmina VidicSusana AmézquetaJasminka GiacomettiSusana FiszmanJasna Canadanovic-BrunetSusanna KugelbergJason SawyerSusumu MuroyaJavier CarballoSuzana Rimac BrnčićJavier FeitoSuzana Rimac-BrnčićJavier MateoSven KarlovićJavier OubiñaSyed Amir AshrafJavier PeñaSyed Imran Ali MeerzaJeaho ChaSylvain HugelJean Daniel CoissonSz-Jie WuJean Daniel CoïssonTae Jin ChoJean Paul DelgadoTaejoon KangJean VanderpasTae-Kyung KimJean-Charles RobinsonTae-Woon KimJean-christophe JacquierTakahiro NodaJeanette K. PurhagenTakashi AmagaiJean-François HocquetteTakashi MaokaJean-Louis LanoiselléTakeshi KatayamaJean-Luc MessionTamas MarosvolgyiJean-Luc PutauxTatiana Souza PortoJean-Marc A. LobaccaroTatsuro OhiraJean-Marc NuzillardTeneva DesislavaJean-Paul VernouxTessa R. EnglundJean-Philippe RalTetsuro AgusaJean-Xavier GuinardThanh-Lam NguyenJeehyun LeeTheofania TsironiJee-Young ImmThomas Kate M.Jeff SavellThomas KotsopoulosJelena KruljThomas Matthew TaylorJelena MijalkovićTibor KovácsJelena MuncanTibor PoósJelena MutićTiffany LegendreJelka PleadinTolulope AshaoluJenifer SantosTomas LafargaJennifer Ahn-JarvisTomáš SadílekJennifer GraefTomohiko IsobeJennifer M. GardnerTomohiko KomatsuJennifer MahonyTony MutukumiraJennifer PerryToribio LauraJens André HammerlTorkmahalleh MehdiAmoueiJens Jørgen SlothTsun-Thai ChaiJens Peter LoyTuba EsatbeyolguJens Petter WoldTusty-Juan HsiehJeong-Hun KangU Gianfranco SpizzirriJeppe Madura LarsenUrania Menkissoglu-SpiroudiJerome MounierVahid FarzanehJerzy GębskiValentina Laghezza MasciJerzy JuśkiewiczVasconi MauroJerzy StangierskiVěra HellerováJesse C. KrakauerVictor LopezJessica WalkerViktoriia V. TorbinaJesús Fernández-LucasVincenzo MingantiJesus Garcia-MadariagaVirginia Celia ResconiJesús Manuel López-BonillaVitali SikirzhytskiJesus Martín-GilVladimír KopeckýJesus OchoaVladimir OstrýJesus ValcarcelVladimír ScholtzJette Feveile YoungWajaht A. ShahJeu-Ming P. YuannWeiwei CuiJheng Hua LinWen-Chien LuJhih-Ying CiouWen-Tzu WuJia XiongWhei-Fen WuJiandong WuWilliam R. NewsonJianming LiuWissem Aidi WannesJian-Ping WuWoei Jye LauJie GaoWoo-jung ParkJihan KimWu HaoJihee KangXiaoli QinJill DieterleXin-Tang LinJim McLauchlinXiuping HeJing LiuYash RavalJing ZhaoYasuyuki MakiJingshu GuoYe-Na KimJinhe BaiYing Chen LuJin-Seng LinYolande T.R. ProrogaJiří KillerYoung Joon SungJi-Ye KeeYuan KimJoachim SchoutetenYueh-Hsia ChiuJoan M. KingYuh-shuen ChenJoan Oñate NarcisoYumin DaiJoana CostaYu-Wei ChangJoana OliveiraYuyun LuJoana SantosZhao ChenJoanna BrysZhenyu ShenJoanna GruszczyńskaZhiyue WangJoanna HarasymZhuohong (Kenny) XieJoanna Kapusta-DuchZiarno MałgorzataJoanna KaszubaZiba BaratiJoanna Kobus-CisowskaZoltán GáspáriJoanna OraczZoltán GyőriJoanna Paciorek-SadowskaZoltán KókaiJoanna Piepiórka-StepukZoltán KovácsJoanna Skopinska-WisniewskaZoltan LaknerJoanna Smoluk-SikorskaZongcai TuJoanna StadnikZuzana HruskaJoanna Suliburska



